# Few-shot learning approach with multi-scale feature fusion and attention for plant disease recognition

**DOI:** 10.3389/fpls.2022.907916

**Published:** 2022-09-16

**Authors:** Hong Lin, Rita Tse, Su-Kit Tang, Zhen-ping Qiang, Giovanni Pau

**Affiliations:** ^1^Faculty of Applied Sciences, Macao Polytechnic University, Macau, Macao SAR, China; ^2^Engineering Research Centre of Applied Technology on Machine Translation and Artificial Intelligence of Ministry of Education, Macao Polytechnic University, Macau, Macao SAR, China; ^3^College of Big Data and Intelligent Engineering, Southwest Forestry University, Kunming, China; ^4^Department of Computer Science and Engineering, University of Bologna, Bologna, Italy; ^5^Samueli Computer Science Department, University of California, Los Angeles, Los Angeles, CA, United States

**Keywords:** few-shot learning, meta-learning, multi-scale feature fusion, attention, plant disease recognition, cross-domain, training strategy, sub-class classification

## Abstract

Image-based deep learning method for plant disease diagnosing is promising but relies on large-scale dataset. Currently, the shortage of data has become an obstacle to leverage deep learning methods. Few-shot learning can generalize to new categories with the supports of few samples, which is very helpful for those plant disease categories where only few samples are available. However, two challenging problems are existing in few-shot learning: (1) the feature extracted from few shots is very limited; (2) generalizing to new categories, especially to another domain is very tough. In response to the two issues, we propose a network based on the Meta-Baseline few-shot learning method, and combine cascaded multi-scale features and channel attention. The network takes advantage of multi-scale features to rich the feature representation, uses channel attention as a compensation module efficiently to learn more from the significant channels of the fused features. Meanwhile, we propose a group of training strategies from data configuration perspective to match various generalization requirements. Through extensive experiments, it is verified that the combination of multi-scale feature fusion and channel attention can alleviate the problem of limited features caused by few shots. To imitate different generalization scenarios, we set different data settings and suggest the optimal training strategies for intra-domain case and cross-domain case, respectively. The effects of important factors in few-shot learning paradigm are analyzed. With the optimal configuration, the accuracy of 1-shot task and 5-shot task achieve at 61.24% and 77.43% respectively in the task targeting to single-plant, and achieve at 82.52% and 92.83% in the task targeting to multi-plants. Our results outperform the existing related works. It demonstrates that the few-shot learning is a feasible potential solution for plant disease recognition in the future application.

## 1. Introduction

Plant disease has always been a significant concern in agriculture since it results in reduction of crop quality and production (Campbell and Madden, 1990; Oerke and Dehne, 2004; Strange and Scott, 2005). Image-based auto-diagnosing method is very accessible and economical for farmers. It is especially friendly to those farmers who are in remote areas or on a small scale. In recent years, deep learning methods are widely used in image-based recognition (Lin et al., 2021). Many networks have achieved excellent performance when trained with relevant large-scale datasets. As we know, the performance of deep learning network relies on data. As the network gets deeper, the number of trainable parameters becomes larger and the demand for data increases. Insufficient data can easily lead to overfitting (Simonyan and Zisserman, 2014; Dong et al., 2021). In plant disease recognition, the existing data resources are limited. Meanwhile, creating a large-scale plant disease dataset is difficult due to: (1) the number of species and diseases are very huge; (2) disease identification and annotation requires expert involvement; (3) some diseases are too rare to collect sufficient samples. The long-tailed distribution of data is common in nature and it is difficult to be used to train a balanced model. In brief, creating large-scale dataset of plant disease is a time-consuming and exhausting work (Deng et al., 2009; Singh et al., 2020). Severe shortage of data has become a barrier to take advantage of deep learning methods.

Generally, there are three ways to alleviate the problems caused by data shortages. Data augmentation, as the most common solution, augments instances by image scaling, rotation, affine transformation, etc. Transfer learning method delivers prior knowledge from source domain to target domain and adapts to the target domain by a small amount of data. But the two solutions cannot generalize to new categories in test, which means that the classes in test must have been learned in training. In addition to these two solutions, meta-learning, an approach that mimics human learning mechanisms, has been proposed in recent years. The objective of this solution is not to learn knowledge, but to learn to learn. Different from the conventional classification methods, few-shot learning (FSL) is a kind of meta-learning method which can quickly generalize to unseen categories with the supports of few samples.

One branch of FSL is metric-based method (Wang et al., 2020). The principle is that the features of samples belonging to the same category are close to each other, while the features of samples belonging to different categories are far from each other. The earliest representative work is Siamese Network, which is trained with positive or negative sample pairs (Koch et al., 2015). Vinyals et al. (2016) proposed the Matching Networks, and they borrowed the concept “seq2seq+attention” to train an end-to-end nearest neighbor classifier. Snell et al. (2017) proposed Prototypical Network, which learns to match the proto center of class in semantic space through few samples. Sung et al. (2018) proposed Relation Network, which concatenates the feature vectors of the support samples and the query samples to discover the relationship of classes. Li et al. (2019) proposed CoveMNet based on the covariance presentation and covariance metric of the consistency of distribution. The network extracts the second order statistic information of each category by an embedding local covariance to measure the consistency of the query samples with the novel classes. Chen et al. (2020) proposed Meta-Baseline method, which achieves good performance on some FSL benchmarks. The accuracy achieves at 83.74% with *5-way, 5-shot* task of Tiered-ImageNet, and 90.95% with *1-way, 5-shot* task of Mini-ImageNet.

Recently, FSL has started to be used in research on plant disease identification. Argüeso et al. (2020) used Siamese Network on the dataset PlantVillage (PV). Jadon (2020) proposed SSM-Net that uses the Siamese framework and combines two features from a Conv and a VGG16. Zhong et al. (2020) proposed a novel generative model for zero-shot and few-shot recognition of citrus aurantium L. diseases by using conditional adversarial auto-encoders. Afifi et al. (2021) compared Triplet network, Baseline, Baseline++, and DAML on PV and coffee leaf datasets. The results show that the Baseline has the best performance. Li and Chao (2021b) proposed a semi-supervised FSL method and tested it with PV. Nuthalapati and Tunga (2021) introduced transformer into plant disease recognition. Chen et al. (2021) used meta-learning on Mini-plant-disease dataset and PV. Li and Yang (2021) used Matching Network and tested cross-domain performance by mixing pest data. These methods have been tried from various perspective and have made important progresses. Nevertheless, FSL still has two common challenging issues: (1) limited features extracted from few samples are less representative for a class (Wang et al., 2020); (2) the generalization requirements are very high and various. In this work, we tackle the two issues by using multi-scale feature fusion (MSFF) and improving training strategies.

CNN is widely used in image-based deep learning methods. In a CNN architecture, the local features with more details and small perceptive fields are extracted from low-level layers, while the global features with rich semantic information and large perceptive fields are extracted from high-level layers (Goodfellow et al., 2016). MSFF is the technology using multi-scale features which are extracted from different layers of CNN (Dogra et al., 2017). In object detection and semantic segmentation, many excellent networks are proposed by using MSFF, such as Feature Pyramid Network (Lin et al., 2017), U-net (Ronneberger et al., 2015), Fully Convolutional Network (Long et al., 2015) etc. MSFF is also used in image restoration, image dehazing and image super resolution etc. (Li et al., 2018; Zhang and Patel, 2018; Zhang et al., 2018; Lan et al., 2020). These methods fuse features by using dense connection, feature concatenation or weighted element-wise summation (Dong et al., 2020). In common, the mentioned methods have encoder-decoder framework. The multi-scale features extracted from encoder are reused in decoder to enhance feature representation. However, in conventional classification task, MSFF is seldom used because the network does not have decoder. Generally, only the top semantic features are fed into classifier, but other scale features are abandoned. But in fact, the high-level features and the low-level features are not subordination relationship. The local features including rich fine-grained features can be an effective compensation to formulate a richer feature representation of sample (Lim and Kang, 2019). In the data-limitation condition, it requires to extract as many features as possible from a limited amount of data. Therefore, in this work, we propose to leverage the MSFF to enhance feature representation. Multi-scale features can be fused in different ways. In our work, we use cascaded multi-scale feature fusion (CMSFF).

The channels of feature maps increase after feature fusion. But it does not mean that all channels are the same significance. The contribution of each channel is different. Some channels should be emphasized and some should be suppressed. Attention can help to focus on the meaningful channels. Attention mechanism plays important role in human perception to selectively focus on salient parts in order to capture visual structure better (Guo et al., 2021). It has been leaded into some areas of machine learning such as computer vision, natural language processing etc. and has significance to improve performance (Hu, 2019; Hafiz et al., 2021). It not only tells where to focus, but also improves the representation of interests. Recently, some light-weight attention modules have been proposed. Wang et al. (2017) proposed Residual Attention Network that uses encoder-decoder style attention module. Hu et al. (2018) introduced a compact module to exploit the inter-channel relationship, which was named as Squeeze-and-excitation module. Woo et al. (2018) proposed Convolutional Block Attention Module that includes channel attention (CA) and spatial attention. These light-weight attention modules can be easily embedded into deep learning networks as plug-ins. In this work, we use the CA to weight the accumulated channels obtained from CMSFF. The CMSFF and CA is an effective combination to enhance the representation of category under few-shot condition.

As the definition of FSL, it is asked to generalize to novel categories or novel domains. Generalizing to new categories within the same domain of training is defined as intra-domain classification, while generalizing to novel domain is defined as cross-domain classification. Long-tail distribution of data is common in plant disease datasets. To identify the part of categories with few samples, the model can be trained with the part of diseases that have more samples. This generalization happens in the same domain. Cross-domain happens when a set of categories with few shots is required to be identified but does not belong to any dataset. Cross-domain adaption happens between different datasets, which is more difficult than intra-domain adaption. However, researchers found that it is frequently encountered situation and inescapable for boosting FSL to practical application. Guo et al. (2020) established a new broader study of cross-domain few-shot learning benchmark and pointed out that all meta-learning methods underperform in relation to simple fine-tuning methods, which indicates that the difficulty of the cross-domain issue. Adler et al. (2020) proposed a method of representation fusion by an ensemble of Hebbian learners acting on different layers of a deep neural network, which is from feature representation perspective. Li W.-H. et al. (2022) proposed a task-specific adapters for cross-domain problem from the perspective of network architecture. Qi et al. (2022) proposed a meta-based adversarial training framework for this problem, which is also from the perspective of network architecture. As we know, there is no research that has been done from a training strategy perspective. These efforts are the kind of general explorations of using general benchmarks (e.g., ImageNet, CIFAR etc.) and rarely discuss specific domains. In fact, different domain has its own characteristics and resources to utilize when crossing domains. Hence, in this work, we propose a set of training strategies to match various cases of generalization using the available data resources.

The contributions of this work are summarized as: (1) we propose a Meta-Baseline (MB) based FSL approach merging with CMSFF and CA for plant disease recognition; (2) we propose a group of training strategies to meet different generalization requirements; (3) through extensive comparative experiments and ablation experiments, we validate the superiority of our method and analyze various factors of FSL. Comparing with the existing related works under the same data conditions, our method has achieved at the best accuracy.

## 2. Materials and methods

### 2.1. Materials

**Figure 1:**
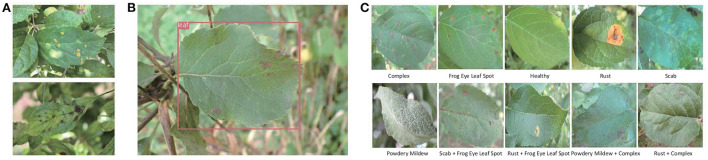
**(A)** The original samples of AFD. **(B)** The leaf detection result by YOLO-v3. **(C)** The samples of 10 classes after segmentation and resizing.

In this research, three public datasets are used in our experiments. Mini-ImageNet is a subset of the ImageNet, which includes 100 classes and 600 images per class. We select 64 classes in our experiments. The second is PV (Hughes and Salathé, 2015) released in 2015 by Pennsylvania State University. It is the most frequently used and comprehensive dataset in academic research up to now in plant disease recognition. Totally, it includes 50,403 images which crosses over 14 crop species and covers 38 classes, as shown in Table 1. Because the number of samples in PV is unbalanced, we use the data after augmentation and select 1,000 images per class to keep balance. The third is the dataset of apple foliar disease (AFD), which was published in FGVC8 Plant Pathology 2021 Competition. All images of AFD were taken in wild with complicated backgrounds, as shown in Figure 1A. We perform pre-processing to reduce the complexity of the surroundings by removing background other than leaves. YOLO-v3 (Redmon and Farhadi, 2018) is adopted to detect leaves in images which is shown in Figure 1B. After segmentation and resizing, the images with a single leaf in each image are used in this work, as shown in Figure 1C.

**Table 1:** The 14 species and 38 categories in PV.

**Species**	**Class number**	**Class name**
Apple	4	Apple scab, black rot, cedar apple rust, healthy
Blueberry	1	Healthy
Cherry	2	Healthy, powdery mildew
Corn	4	Gray leaf spot, common rust, healthy, northern leaf blight
Grape	4	Black rot, black measles, healthy, leaf blight
Orange	1	Haunglongbing
Peach	2	Bacterial spot, healthy
Pepper	2	Bacterial spot, healthy
Potato	3	Early blight, healthy, late blight
Raspberry	1	Healthy
Soybean	1	Healthy
Squash	1	Powdery mildew
Strawberry	2	Healthy
Tomato	10	Bacterial spot, early blight, healthy, late blight, leaf mold, septoria leaf spot, spider mites, target, mosaic virus, yellow leaf curl virus

The hardware configurations are: Graphics: Tesla V100-DGXS-32GB; Video Memory: 32*G*×4; Processor: Intel(R) Xeon(R) CPU E5-2698 v4 @ 2.20GHz; Operating System: Ubuntu 18.04.6 LTS.

### 2.2. Problem formulation

In FSL paradigm, given two labeled sets with categories *C*_*train*_ and *C*_*novel*_, *C*_*train*_ is used in training and *C*_*novel*_ is used in test. The two sets are exclusive, *C*_*train*_∩*C*_*novel*_ = ∅, which means that categories used in test are not seen during training. Data is formulated to tasks and each task *T* is made up of a *supportsetS* and a *querysetQ*. The sample of *S* is denoted by (*x*_*s*_, *y*_*s*_) which is a (*image, label*) pair and the sample of *Q* is denoted by (*x*_*q*_, *y*_*q*_). In training, the label *y*_*q*_ is used for calculating loss, which is supervised learning.

An *N-way, K-shot* task indicates that the *S* contains *N* categories with *K* samples in each category, and the *Q* contains the same *N* categories with *W* samples in each category. The goal is to classify the *N*×*W* unlabeled samples of Q into *N* categories. For evaluation, the average accuracy is computed from many tasks sampled from *C*_*novel*_, *N* ∈*C*_*novel*_.

### 2.3. Architecture

#### 2.3.1. Meta-Baseline framework

**Figure 2:**
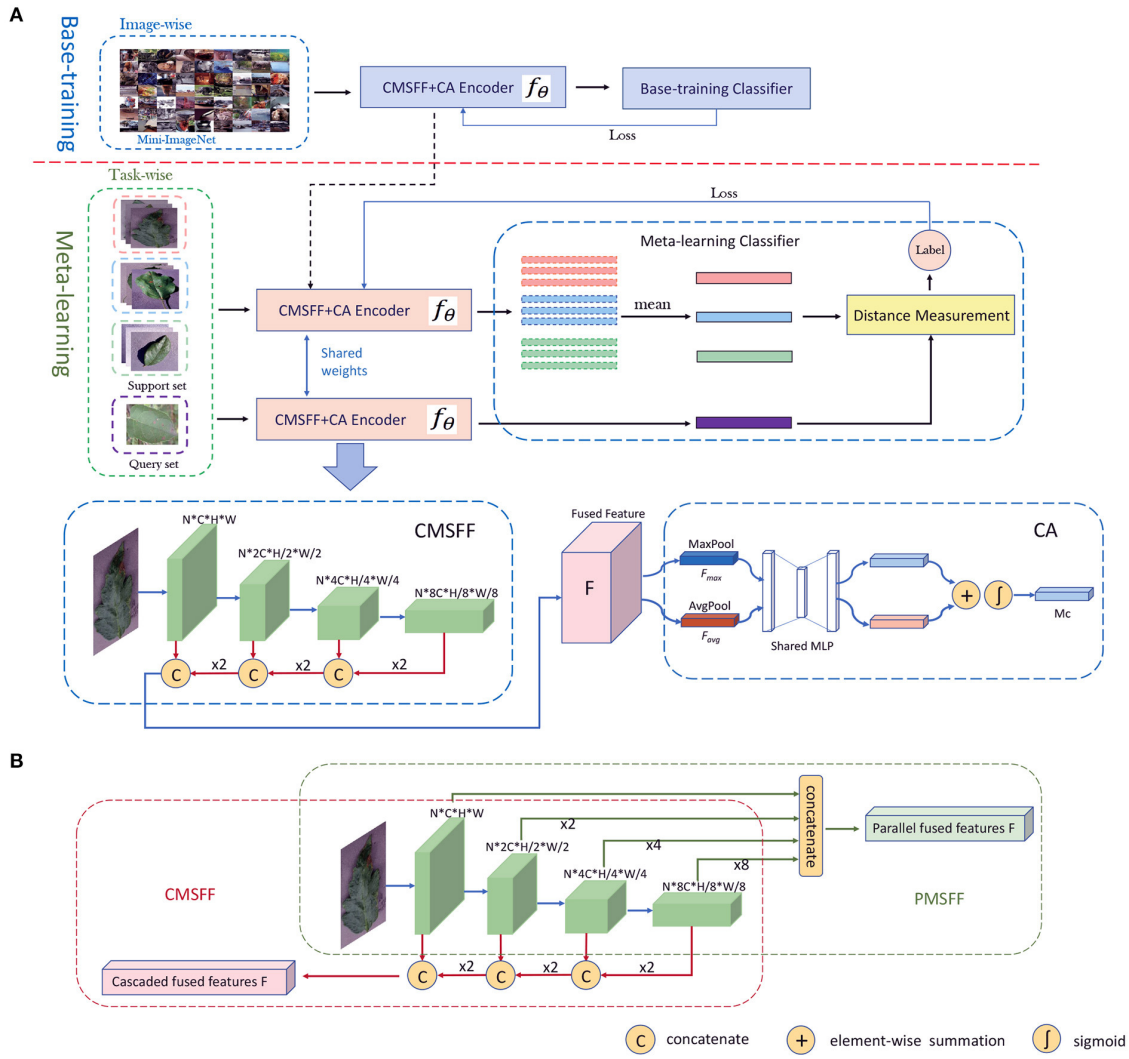
**(A)** The network architecture of our method. The training includes two stages: base-training stage and meta-learning stage. The CMSFF+CA Encoder is unfolded to CMSFF module and CA module. **(B)** The parallel multi-scale feature fusion and cascaded multi-scale feature fusion.

Like classical classification structure, our framework contains two components: an encoder and a classifier, which is illustrated in Figure 2A. The encoder noted as *f*_θ_ is a CNN-based network merging with CMSFF and CA. It is trained in two stages: base-training and meta-learning.

In base-training, the network contains *f*_θ_ and base-training classifier, which is trained with image-wise data. The goal in this stage is to learn the general features as prior knowledge. Some large-scale general datasets with more classes and diverse data, such as ImageNet, Mini-ImageNet etc. are good choices for learning prior knowledge. The classifier can be linear classifier, fully connected layer, SVM, or other classifiers. The cross-entropy loss is calculated to update the parameters of *f*_θ_ during back propagation. After base-training is completed, the classifier is removed and the trained model is delivered to the meta-learning stage.

In meta-learning, *f*_θ_ is initialized by the trained model from base-training. Meta-learning is a concept of learning to learn. So, the purpose is not to learn the knowledge of the training classes, but to learn how to differentiate between classes. Aiming at the objective, the classifier in meta-learning is replaced by a distance measurement module. The classification result is decided by the distances from the support samples to the query sample. Meta-learning is a task-driven paradigm where training data is formulated as *N-way, K-shot* tasks. Based on a simple machine learning principle: test and training conditions must match (Vinyals et al., 2016), the data of *C*_*novel*_ is also formatted into tasks in test.

Given an *N-way, K-shot* task, *K* samples of a category *c* in *S* are embedded into feature space by *f*_θ_ and become *K* feature vectors. A mean vector of the *K* vectors are calculated as the centroid of *c*, which is consider as the representative of category *c*:


(1) $$\begin{array}{l} \omega_{c}=\frac{1}{\left|S_{c}\right|}\underset{x_{s}\in S_{c}}{\sum }f_{\theta }\left(x_{s}\right) \end{array}$$

where, *S*_*c*_ denotes the samples of class *c* in *S*, |*S*_*c*_| = *K*, *x*_*s*_ denotes each sample of class *c*. The query sample *x*_*q*_ in an *N-way, K-shot* task is also embedded by *f*_θ_. The probability that sample *x*_*q*_ belongs to class *c* is calculated as:


(2) $$\begin{array}{l} p(y=c|x_{q})=\frac{exp(\gamma .<f_{\theta }(x_{q}),\omega_{c}>)}{\sum_{c^{\prime }}exp(\gamma .<f_{\theta }(x_{q}),\omega_{c^{\prime }}>)} \end{array}$$

where, < ., .> denotes the distance of two vectors, *c*′ denotes all the classes in *S*, $\omega_{c^{\prime }}$ denotes all the centroids of *S*, γ is a learnable parameter to scale the distance. In training, we use cross-entropy loss to update the parameters of the network. The algorithm of meta-learning is shown in Table 2.

**Table 2:** The algorithm of meta-learning.

**Algorithm of meta-learning**
***Input*****:** data_loader,n_way,n_shot,n_query,task_per_batch
***Output*****:** avg_acc, avg_loss
**for** i in epoch:
*train*:
**for** j in batch:
*task* = *task*(*data*_*loader, n*_*way, n*_*shot, n*_*query, task*_*per*_*batch*)
*x*_0_···*x*_*n*_ = *f*_θ_(*task*.*x*_*shot*)
*x* = *mean*(*x*_0_···*x*_*n*_)
*y* = *f*_θ_(*task*.*x*_*query*)
*logits* = *classifier*(*distance*(*x, y*))
*loss* = *cross*_*entropy*(*logits, task*.*label*)
*acc* = *compute*_*acc*(*logits, task*.*label*)
*loss*.*backwardpropagation&optimize*
**end for**
*validation*:*val*
*compute*:*avg*_*acc, avg*_*loss*
**end for**
***return*****:** avg_acc, avg_loss

#### 2.3.2. Distance measurement

After embedding, the 2D color image has been a high dimensional vector in semantic space. The distance of query sample to the class centroid is calculated by a distance metric. Distance metric uses distance function which provides a relationship metric between each element in the dataset. In many machine learning algorithms, distance metric is used to know the input data pattern in order to make any data-based decision. The most common used measures to calculate the distance between two vectors are cosine similarity, dot product and Euclidean distance.

Cosine similarity is a measure of similarity between two non-zero vectors of an inner product space. It is measured by the cosine of the angle between two vectors and determines whether two vectors are pointing in roughly the same direction. It is the same as the inner product after normalization (Han et al., 2012). In Euclidean geometry, the dot product of the Cartesian coordinates of two vectors is widely used. It is often called as inner product or projection product of Euclidean space. The length of projection represents the distance of two vectors. In mathematics, the Euclidean distance between two high-dimensional vectors is the square root of the sum of the squares of the distances in each dimension.

#### 2.3.3. MSFF

Basically, the structure of MSFF includes two categories: parallel multi-scale feature fusion (PMSFF) and cascaded multi-scale feature fusion (CMSFF). The two fusion methods are illustrated in Figure 2B. The PMSFF concatenates the features from different layers of CNN simultaneously. The different resolutions of feature maps are uniformed before concatenation. Comparatively, the CMSFF fuses the different resolution feature maps step by step. Taking Resnet12 as backbone network, four convolutional blocks are linked. A group of feature maps of double times of channels and half resolution is generated after each block forwarding. In the backward fusion, small size feature maps are two times up-sampled and concatenated with the feature maps of previous block. After a series of up-sampling and concatenation, all channels are fused together to be the fused full-scale feature, noted as *F*. The CMSFF is used in this work.

#### 2.3.4. CA

The CA is used to exploit the inter-channel relationship of features by learning the weights of channels (Woo et al., 2018). The structure of CA module is shown in Figure 2B. Each channel of *F* is considered as a feature detector. The spatial dimension of input feature map is aggregated by pooling operation. In this module, average-pooling and max-pooling are conducted simultaneously and two spatial context descriptors: *F*_*avg*_ and *F*_*max*_, are generated, respectively. Then they are forwarded to a shared network which is composed of multi-layer perceptron (MLP) with one hidden layer. The element-wise summation of the two outputs from MLP goes through a sigmoid. Then the channel attention map $M_{c}\in {\mathbb{R}}^{C\times 1\times 1}$ is produced.

## 3. Results

We carried out 43 groups of comparison experiments and ablation experiments to illustrate our method, training strategies, and the effects of various factors. The details of experiments and results are illustrated and analyzed as below. The bold values listed in tables indicate the highest results for each group under the same conditions.

### 3.1. Data settings

**Figure 3:**
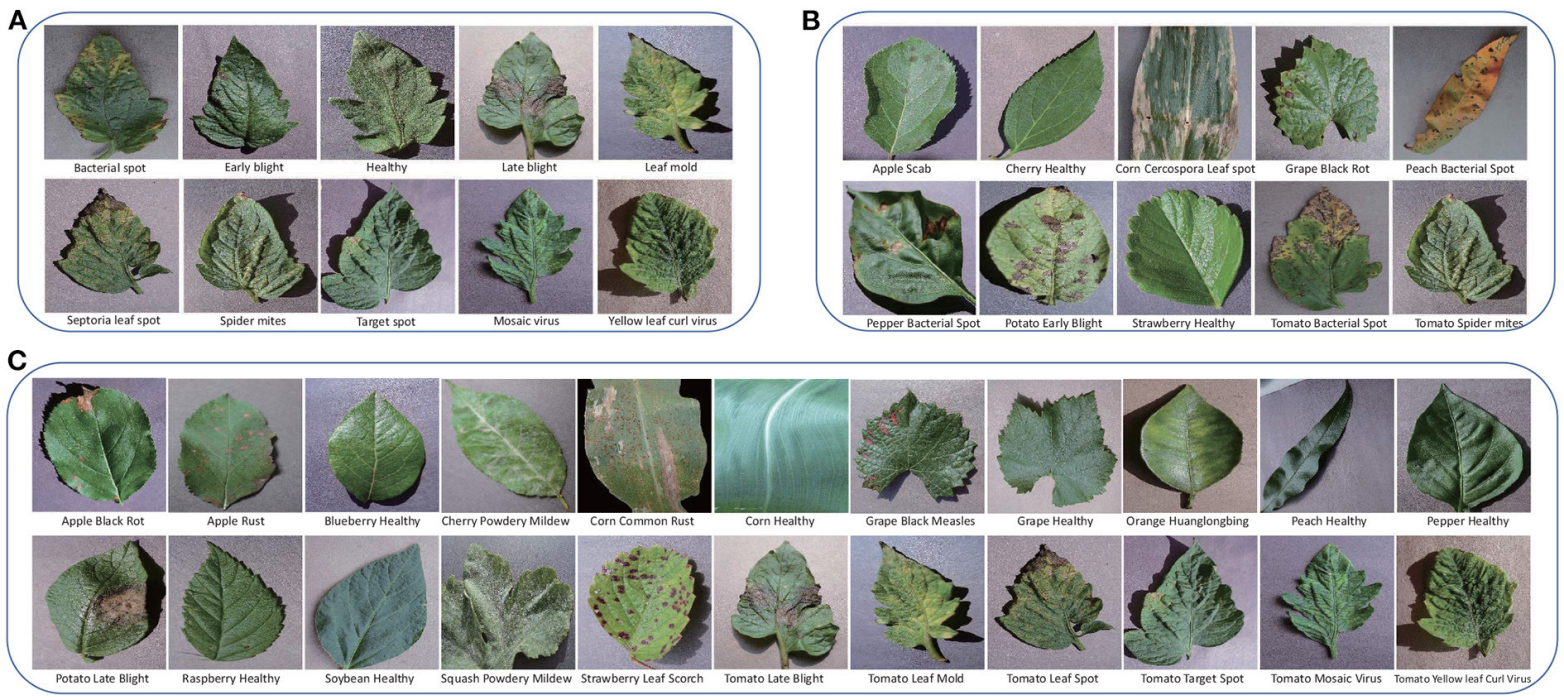
**(A)** The testing classes of PV-Setting-1. **(B)** The testing classes of PV-Setting-2. **(C)** The testing classes of PV-Setting-3.

The PV is separated into three parts for training, validation, and test, respectively. According to the requirement of FSL: the testing categories are novel, the classes of the three parts do not intersect, *C*_*train*_∩*C*_*val*_∩*C*_*test*_ = ∅. In this work, PV is split to three settings as shown in Table 3. PV-Setting-1 is with 22 classes for training, 6 classes for validation, and 10 classes covered by tomato for test. The samples are shown in Figure 3A, which are very similar with each other. PV-Setting-2 is with 22 classes for training, six classes for validation, and 10 classes belonging to nine different species for test. The samples of this setting are shown in Figure 3B. PV-Setting-3 exchanges the training set and testing set of PV-Setting-1 and keeps the same validation set as PV-Setting-1, using 10 classes for training and 22 classes for test. The samples are shown in Figure 3C. The three settings represent “sub-class” task, “train more, test less” task and “train less, test more” task, respectively. In addition, 10 classes of AFD and 200 samples per class are used in this work for cross-domain testing purpose. Since all classes belong to the same super-class: apple leaf, it is also a sub-class classification task.

**Table 3:** Three data settings of PV used in our experiments.

**ID**	**Training**	**Validation**	**Test**
PV-Setting-1 (22-6-10)	(PV-1-22): apple-3,blueberry-1,cherry-2,corn-3,grape-3,orange-1,peach-2,pepper-1,potato-2,raspberry-1,soybean-1,squash-1,strawberry-1	Apple-1,corn-1,grape-1,pepper-1,potato-1,strawberry-1	(PV-1-10T): tomato-10
PV-Setting-2 (22-6-10)	(PV-2-22): apple-2,blueberry-1,cherry-1,corn-2,grape-2,orange-1,peach-1,pepper-1,potato-1,raspberry-1,soybean-1,squash-1,strawberry-1,tomato-6	Apple-1,corn-1,grape-1,potato-1,tomato-2	(PV-2-10): apple-1,cherry-1,corn-1,grape-1,peach-1,pepper-1,potato-1,strawberry-1,tomato-2
PV-Setting-3 (10-6-22)	(PV-3-10): apple-1,cherry-1,corn-1,grape-1,peach-1,pepper-1,potato-1,strawberry-1,tomato-2	Apple-1,corn-1,grape-1,potato-1,tomato-2	(PV-3-22): apple-2,blueberry-1,cherry-1,corn-2,grape-2,orange-1,peach-1,pepper-1,potato-1,raspberry-1,soybean-1,squash-1,strawberry-1,tomato-6

The total 38 classes are separated into three parts for training, validation and test, respectively. “Apple-1” means a class of apple species.

### 3.2. Training strategy

The domain of training is noted as source domain (SD), and the domain of test is noted as target domain (TD). Data from different domains can be used in the three stages: base-training, meta-learning, and test. It is special that there are two training stages of our method, and the datasets used in the two stages could be different. We just consider the domain of meta-learning stage as SD. When SD is the same as TD, it is intra-domain adaption, otherwise, it is cross-domain adaption.

**Figure 4:**
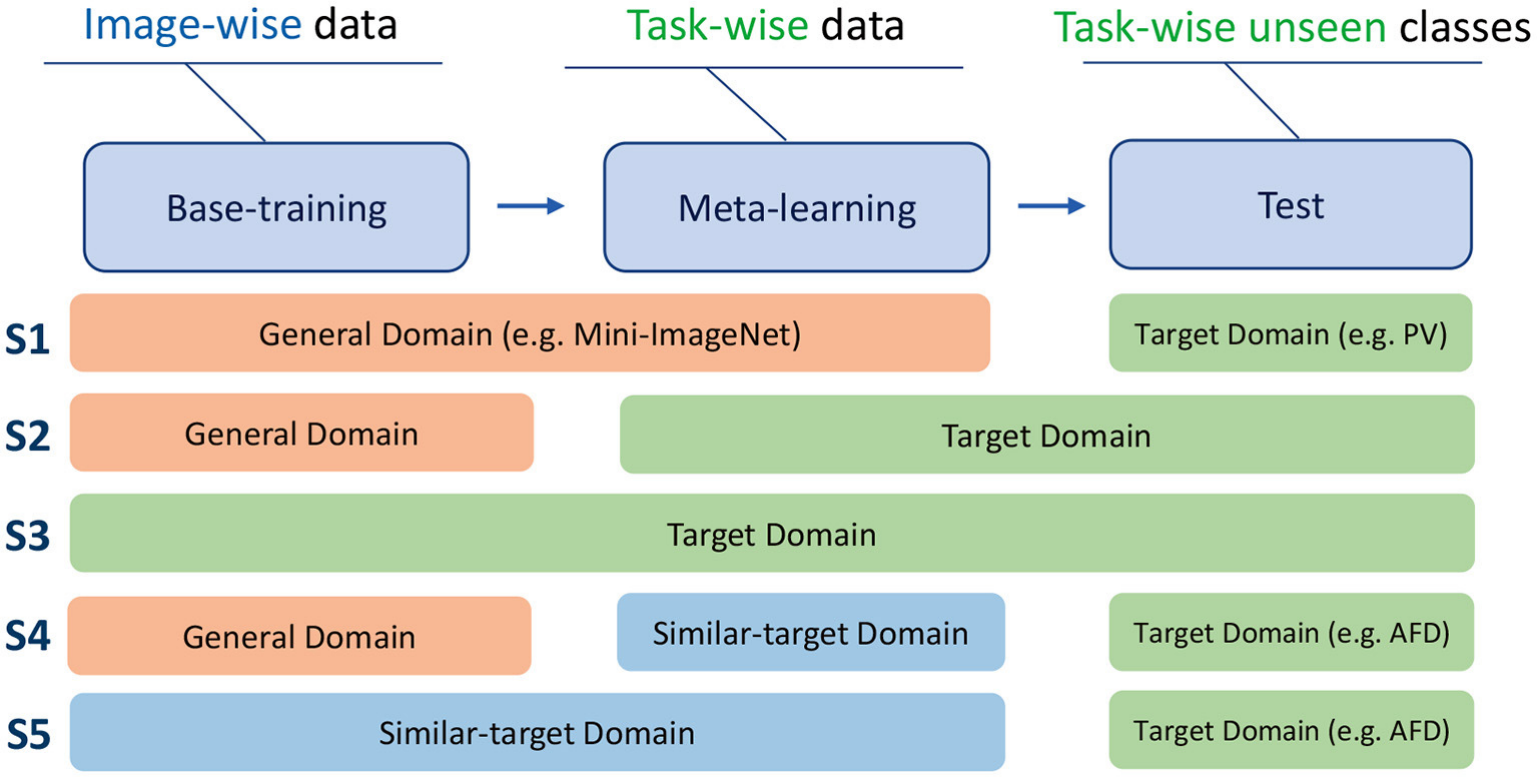
The data formats used in base-training, meta-learning, and test. The five training strategies.

In order to mimic different adaption situations, we design different data configurations. Five adaption configurations using Mini-ImageNet, three PV settings, and AFD are proposed. As shown in Figure 4, *S*1 uses a general dataset (e.g., Mini-ImageNet) in base-training and meta-learning, then uses target dataset (e.g., PV) in test, which is the adaptation from one domain to another, denoted in Formula 3. *S*2 uses a general dataset in base-training, target dataset in meta-learning and test, which is denoted in Formula 4. *S*3 uses target dataset in three stages, which is denoted in Formula 5. *S*4 uses general dataset in base-training, similar-target dataset (e.g., PV) in meta-learning, and target dataset (e.g., AFD) in test, which is denoted in Formula 6. When AFD is used in test, PV is considered as a similar domain as the target domain, because they are both associated with leaf diseases of the plants. *S*5 uses the similar-target dataset in base-training and meta-learning, and target domain dataset in test, which is denoted in Formula 7. *S*1, *S*4, *S*5 are cross-domain, and *S*2, *S*3 are intra-domain.


(3) $$\begin{array}{l} S1:G\to G\to T \end{array}$$

(4) $$\begin{array}{l} S2:G\to T\to T \end{array}$$

(5) $$\begin{array}{l} S3:T\to T\to T \end{array}$$

(6) $$\begin{array}{l} S4:G\to S\to T \end{array}$$

(7) $$\begin{array}{l} S5:S\to S\to T \end{array}$$

where, *G* denotes the general domain, T denotes the target domain, *S* denotes the similar-target domain.

As shown in Table 4, e1, e2, and e3 are conducted with Mini-ImageNet and PV-Setting-1 by using *S*1, *S*2, *S*3. e4, e5, e6 are conducted with Mini-ImageNet and PV-Setting-2 by using *S*1, *S*2, *S*3. e7, e8, e9 are conducted with Mini-ImageNet and PV-Setting-3 by using *S*1, *S*2, *S*3. e10, e11, and e12 are conducted with Mini-ImageNet, PV-Setting-2, and AFD by using *S*1, *S*4, *S*5. For the 12 experiments, the training epoch is 100, and the learning rate is 0.1 and decayed to 0.01 after 90 epochs in base-training. In meta-learning, the training epoch is 50, and the learning rate is 0.001. The validation task is 5-*way*, 1-*shot*, 15-*query*. The backbone network is Resnet12. The distance metric is cosine similarity.

**Table 4:** The group of experiments with different training strategies and different data settings.

**ID**	**Method**	**TS**	**Base-training**	**Meta-learning**	**Test**	**1-shot**	**5-shot**	**10-shot**	**20-shot**	**30-shot**	**40-shot**	**50-shot**
**PV-Setting-1**										
e1	MB	S1	Mini	Mini	PV-1-10T	41.08	60.59	66.27	69.87	71.26	71.86	72.30
e2	MB	S2	Mini	PV-1-22	PV-1-10T	56.07	72.90	76.62	78.87	79.74	79.81	80.11
e3	MB	S3	PV-1-22	PV-1-22	PV-1-10T	**57.85**	**75.04**	**79.08**	**81.51**	**82.47**	**82.83**	**83.08**
**PV-Setting-2**										
e4	MB	S1	Mini	Mini	PV-2-10	60.23	83.08	87.02	88.97	89.61	89.76	90.12
e5	MB	S2	Mini	PV-2-22	PV-2-10	80.88	**91.75**	**93.44**	**94.27**	**94.53**	**94.70**	**94.84**
e6	MB	S3	PV-2-22	PV-2-22	PV-2-10	**81.05**	91.47	93.14	94.00	94.29	94.41	94.53
**PV-Setting-3**										
e7	MB	S1	Mini	Mini	PV-3-22	65.46	85.37	88.81	90.54	91.09	91.33	91.45
e8	MB	S2	Mini	PV-3-10	PV-3-22	**78.74**	**88.96**	**90.58**	**91.52**	**91.97**	**92.05**	**92.17**
e9	MB	S3	PV-3-10	PV-3-10	PV-3-22	74.58	84.77	86.82	87.82	88.29	88.43	88.57
***AFD***							
e10	MB	S1	Mini	Minit	AFD-10	28.26	39.12	44.20	47.83	49.02	50.31	51.32
e11	MB	S4	Mini	PV-2-22	AFD-10	**38.41**	**51.71**	**55.58**	**58.08**	**58.84**	**59.70**	**60.09**
e12	MB	S5	PV-2-22	PV-2-22	AFD-10	36.19	49,16	54.05	57.13	58.47	59.25	59.46

(Task in meta-learning: 5-way, 1-shot, 15-query; backbone network: Resnet12; batchsize: 128; Lr: 0.1 in base-training, 0.001 in meta-learning; distance metric: cosine similarity; Mini, Mini-ImageNet; TS, training strategy).

#### 3.2.1. Intra-domain

**Figure 5:**
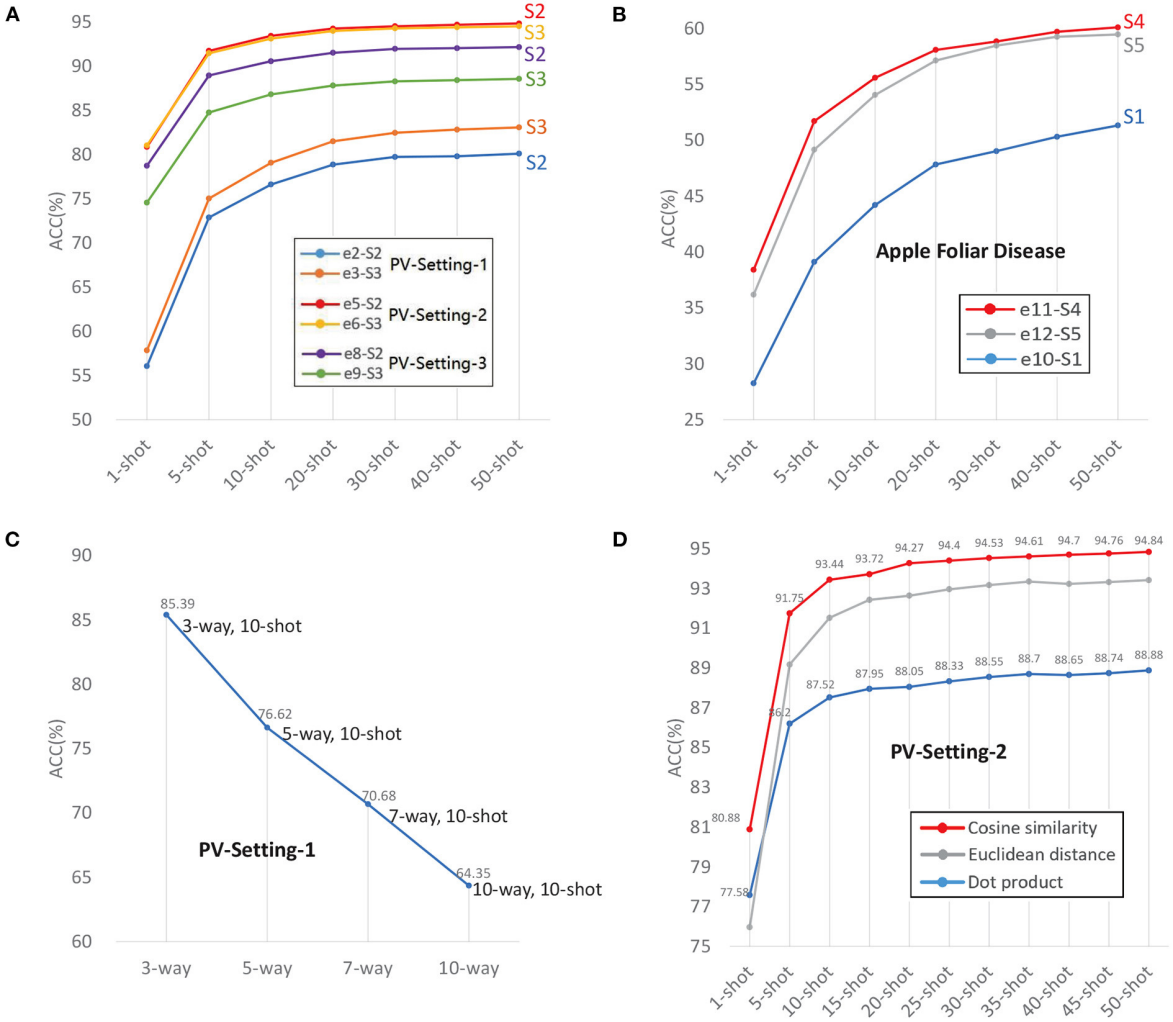
**(A)** Intra-domain experiments with three data settings. **(B)** Cross-domain experiments with AFD. **(C)** The accuracy decreases as Way increases. **(D)** Distance metrics.

According to the definitions of SD and TD, e2, e3, e5, e6, e8, e9 are intra-domain experiments, because the data used in meta-learning and test is from the same dataset. The results are shown in Table 4 and Figure 5A. In PV-Split-2, the accuracy of e5 is better than e4 and e6. In PV-Split-3, the accuracy of e8 is better than e7 and e9. What the two settings have in common is that the disease classes belong to different plants. To the diverse species cases, *S*2 is better than *S*1 and *S*3. Especially when the number of species is bigger, the superiority of *S*2 is more obvious. As listed, e6 gets close to e5, but e8 is much better than e9, which means that the general dataset is better supported when the testing data is more diverse. A broad prior knowledge is very useful for adapting to diverse target. However, in PV-Split-1, e3 is the best one by using *S*3 because the testing data belongs to the same plant. So, the features of testing data are intensive and the general date in base-training is not helpful. Oppositely, the data belonging to the same dataset is easier for adaption. In short, to the intra-domain cases, if the testing classes are of super-classes, *S*2 is the best strategy. If the testing classes are sub-classes, *S*3 is the best strategy.

#### 3.2.2. Cross-domain

Experiments e1, e4, e7, e10, e11, e12 are cross-domain cases. e1, e4, e7, e10 are the experiments with the worst results in their respective data settings by using *S*1, due to the big gap between the general domain and target domain.

Comparing e10, e11 and e12, e11 has the highest accuracy by using *S*4, which are shown in Table 4 and Figure 5B. e12 is not as good as e11 because too intensive features extracted from monotonous samples leads to weaker adaptation. *S*4 is the best training strategy for cross-domain cases, which uses general dataset in base-training to learn the prior knowledge in a wide range, and uses similar-target dataset in meta-learning for adapting to new domain smoothly.

### 3.3. CMSFF and CA

Ablation experiments e13–e22 are conducted to show the positive effects of CMSFF module and CA module, respectively. The results are listed in Table 5. Under four data configurations: PV-Setting-1, PV-Setting-2, PV-Setting-3, and AFD, we execute 8 experiments. The training settings are listed: Mini-ImageNet is used in base-training; backbone network is Resnet12; distance metric is cosine similarity; training strategy is *S*2 and *S*4. Taking e2, e5, e8, e11 as the baseline, the CMSFF module is added and the results of e13, e15, e19, e21 show the improvement of CMSFF. e14, e18, e20, and e22 indicate that CA has further improved the performances on the basis of CMSFF. e15 and e17 are used to compare the PMSFF module with the CMSFF module, and the results show that CMSFF outperforms PMSFF.

**Table 5:** The ablation experiment results of MB, MB+CMSFF, and MB+CMSFF+CA.

**ID**	**Method**	**TS**	**1-shot**	**5-shot**	**10-shot**	**20-shot**	**30-shot**	**40-shot**	**50-shot**
**PV-Setting-1**									
e2	MB	S2	56.07	72.90	76.62	78.87	79.74	79.81	80.11
e13	MB+CMSFF	S2	61.20	77.09	80.92	83.03	84.05	84.34	84.56
e14	MB+CMSFF+CA	S2	**61.24**	**77.43**	**81.28**	**83.59**	**84.46**	**84.70**	**84.86**
**PV-Setting-2**									
e5	MB	S2	81.05	91.47	93.14	94.00	94.29	94.41	94.53
e15	MB+PMSFF	S2	81.46	91.86	93.51	94.57	94.81	94.88	95.03
e16	MB+CMSFF	S2	82.21	92.32	93.87	94.71	95.03	95.15	95.31
e17	MB+PMSFF+CA	S2	81.87	92.39	93.93	94.86	95.29	95.31	95.50
e18	MB+CMSFF+CA	S2	**82.52**	**92.83**	**94.39**	**95.29**	**95.65**	**95.73**	**95.74**
**PV-Setting-3**									
e8	MB	S2	74.58	84.77	86.82	87.82	88.29	88.43	88.57
e19	MB+CMSFF	S2	76.61	88.45	90.17	91.32	91.78	91.86	92.14
e20	MB+CMSFF+CA	S2	**78.15**	**89.57**	**91.24**	**92.46**	**92.67**	**93.02**	**93.07**
**AFD**									
e11	MB	S4	38.41	51.71	55.58	58.08	58.84	59.70	60.09
e21	MB+CMSFF	S4	40.77	54.14	57.68	60.13	61.30	62.03	62.69
e22	MB+CMSFF+CA	S4	**43.94**	**56.93**	**60.64**	**63.66**	**64.50**	**65.55**	**66.18**

(Base-training: Mini-ImageNet; backbone network: Resnet12; distance metric: cosine similarity; TS, training strategy).

### 3.4. Sub-class classification

Sub-class is defined as the classes belong to the same entry class. The PV-Setting-1 and AFD are sub-class classification examples. Sub-class classification is also named as fine-grained vision categorization which aims to distinguish subordinate categories within entry level categories. Because the samples belonging to the same super-class are similar with each other, sub-class classification is a challenging problem.

In Table 4, the PV-setting-1 is the lowest accuracy group among the three PV-settings, as the samples all belong to tomato and are indistinguishable. The results of AFD group are worse than PV-Setting-1, which is not only because of Sub-class reason, also due to cross-domain and in-wild setting of images. Even if the images of AFD are already pre-processed, the backgrounds of images are still different from PV. Also, the illumination condition, resolution, photography devices are all different. Intuitively, the gap of features from SD to TD causes the accuracy declining.

### 3.5. Way and shot

*N-way* and *K-shot* are the configurations of the task that indicate the difficulty of the task. Given a fixed *K*, the accuracy decreases as *N* increases. The result of PV-split-1 with *N-way, 10-shot* is shown in Figure 5C. The accuracy drops down from 85.39% to 64.35% as *N-way* increases from 3 to 10.

All experimental results listed in Table 4 are executed with fixed *5-way*, which indicates that regardless of the data configurations, all experiments follow the common trend: accuracy increases with the number of shots. The accuracy sharply increases as the *Shot* increases from *1-shot* to *5-shot*, and tends to be stable when the *Shot* is larger than 10. After the *shot* is larger than 20, the growth is not significant. From *1-shot* to *50-shot*, the increase of accuracy ranges from at least 10% to a maximum of 32%.

The results show that the accuracy increases with the number of *shot* and decreases with the number of *way*. More *ways* means higher complexity, and more *shots* means more supporting information. In existing researches, the *N*−*way* is set to 5 generally. In application scenarios, the *N* is determined by the number of target categories and should not be limited to 5. For example, a plant may have more than five diseases, then the *ways* should the same as the number of diseases that may occur in the specific scenario. *N-way* and *K-shot* are a pair with trade-off relationship. When expanding novel classes, we can increase the number of shots as compensation to maintain accuracy. For a new class to be identified, it is acceptable to collect 10 to 50 samples as its support set. However, the positive relationship of shots and accuracy is not linear. The increase of accuracy as *K-shot* has ceiling. When the *K* is larger than 30, the accuracy is still growing but very slowly.

### 3.6. The diversity of meta-learning data

The number of classes in meta-learning is noted as *N*_*train*_, and noted as *N*_*test*_ in test. Comparing e5 with e8, they are both trained with Mini-ImageNet in base-training. e5 uses 28 classes in meta-learning and 10 classes in test, which is the case *N*_*train*_>*N*_*test*_. The training set and testing set of e5 are exchanged in e8, which is the case *N*_*train*_<*N*_*test*_.

The training tasks and testing tasks are all formulated as *5-way*, which means that five classes are sampled in each task. The *N*-*way* of task is the same in e5 and e8. However, the accuracy of e5 is at least 2% higher than e8. It indicates that the size of data used in meta-learning is a factor effects the performance. Using more classes in meta-learning leads to positive results, providing more diverse features and improving the robustness of the model.

### 3.7. Distance metric

In this work, we compared three distance metrics: dot product, cosine similarity, and Euclidean distance. The same distance measurement module is used in meta-learning and test. This is because even if there is no parameter to be trained in this module, the losses calculated from the distance measurement still affect the parameter updates in the iterations.

An appropriate distance metric significantly helps in improving the performance of classification, clustering process etc. Cosine similarity hits the best performance, as shown in Table 6 and in Figure 5D. The reason is that the vectors obtained from encoder are high dimensional vectors. The cosine similarity has often been used to counteract the problem of Euclidean distance in high dimensional space. The normalization in cosine similarity also has positive effect.

**Table 6:** The results of different distance metrics.

**ID**	**Metric**	**1-shot**	**5-shot**	**10-shot**	**20-shot**	**30-shot**	**40-shot**	**50-shot**
e23	Dot product	77.58	86.2	87.52	88.05	88.55	88.65	88.88
e5	Cosine similarity	**80.88**	**91.75**	**93.44**	**94.27**	**94.53**	**94.70**	**94.84**
e24	Euclidean distance	75.96	89.17	91.52	92.64	93.17	93.23	93.42

(Method: MB; backbone network: Resnet12; batchsize: 128; Lr: 0.1 in base-training, 0.001 in meta-learning).

### 3.8. Backbone networks

In this work, we compared different backbone networks: Convnet4 (Snell et al., 2017), AlexNet (Krizhevsky et al., 2012), Resnet12, Resnet18, Resnet50, Resnet101 (He et al., 2016), DenseNet (Huang et al., 2017), MobileNet-V2 (Sandler et al., 2018). The Convnet4 is the classical architecture used in FSL which stacks four blocks of convolutional calculation. Different networks include different sizes of trainable parameters. The trainable parameters are more in base-training than in meta-learning because the base-training classifier is removed in meta-learning. The size of trainable parameters, learning rate (Lr), training time, and epochs in the two training stages are listed in Table 7. e25–e31 are conducted with the configuration: Mini-ImageNet is used in base-training (100 epochs) and PV-2-22 is used in meta-learning. The different number of iterations is due to the different convergence speed in meta-learning. The performances of the backbone networks are listed in Table 8. Resnet12 and Resnet50 outperform the other networks, with Resnet12 being more efficient.

**Table 7:** The experiment efficiencies of different backbone networks.

		**Base-training**					**Meta-learning**			
**ID**	**Backbone network**	**Size**	**Lr**	**Training time**	**Epoch**		**Size**	**Lr**	**Training time**	**Epoch**
e25	Convnet4	215.6 K	0.01	40 m	100		113.1 K	0.001	31 m	50
e26	AlexNet	3.8 M	0.01	40 m	100		3.7 M	0.001	17 m	50
e5	Resnet12	8.0 M	0.1	1.2 h	100		8.0 M	0.001	18 m	20
e27	Resnet18	11.2 M	0.1	1.4 h	100		11.2 M	0.001	40 m	50
e28	Resnet50	23.6 M	0.1	2.3 h	100		23.5 M	0.001	38 m	30
e29	Resnet101	42.6 M	0.01	3.3 h	100		42.5 M	0.001	35 m	20
e30	DenseNet	791.1 K	0.1	3.8 h	100		769.2 K	0.001	1.9 h	50
e31	MobileNet-v2	3.6 M	0.1	2.2 h	100		3.5 M	0.001	1.0 h	50

(Bae-training: Mini-imageNet; meta-learning: PV-2-22; distance metric: cosine similarity).

**Table 8:** The results of different backbone networks.

**ID**	**Backbone networks**	**1-shot**	**5-shot**	**10-shot**	**20-shot**	**30-shot**	**40-shot**	**50-shot**
e25	Convnet4	69.06	85.91	89.91	91.88	92.35	92.79	93.11
e26	AlexNet	68.35	83.12	85.73	87.00	87.27	87.44	87.92
e5	Resnet12	80.88	**91.75**	**93.44**	**94.27**	**94.53**	**94.70**	**94.84**
e27	Resnet18	78.58	89.16	91.36	91.96	92.26	92.44	92.78
e28	Resnet50	**80.89**	90.91	92.56	93.86	94.08	94.15	94.33
e29	Resnet101	74.93	85.59	87.63	89.12	89.67	89.91	89.91
e30	DenseNet	79.39	89.21	90.82	91.84	92.21	92.10	92.50
e31	MobileNet-V2	78.17	89.21	91.48	92.42	92.83	93.02	93.41

(Method: MB; backbone network: resnet12; batchsize: 128; Lr: 0.1 in base-training, 0.001 in meta-learning; Data: Mini-imageNet in base-training, PV-setting-2 in meta-learning and test).

**Figure 6:**
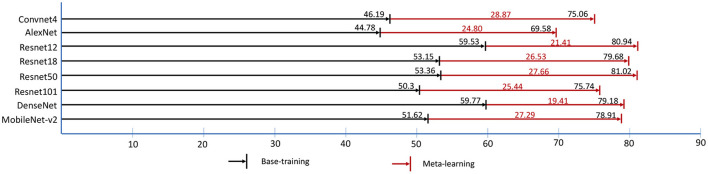
The best validation accuracy (%) of “1-shot, 5-way” task in base-training and meta-learning. The red digits represent the accuracy lifting ranges (%) of meta-learning.

In base-training and meta-learning, we use the validation data to test the accuracy of *5-way, 1-shot* tasks which is shown in Figure 6. The black numbers on the black lines are the best accuracy in base-training, and the black numbers on the red lines are the best accuracy in meta-learning. The lifting ranges of accuracy in meta-learning are marked in red numbers. It is shown that the model trained in base-training stage already has the identification ability with few shots to some extent, even without training with tasks in meta-learning. However, in base-training, the model is already convergent by training with image-wise data, and the accuracy of task testing no longer increases. In fact, the model still has space to improve. Based on this, in meta-learning, by using task-wise data, the accuracy has been further promoted around 20% to 30%.

In recent years, the architectures of networks go deeper and deeper. Some researchers proposed a question that do we really need so deep networks? Our results show that a medium-sized network outperforms other networks in this task. We summarized two reasons: (1) In CNNs, the simpler and more basic features are learnt in shallower layers, the more abstract and complex features are learnt from deeper layers. From shallower layers to deeper layers, the features transition from edges, lines, and colors, to textures and patterns, to complex graphics, even to specific objects. For our specific task, even humans (e.g. plant experts) rely more on color, shape, and texture for disease identification. Hence, the too deep networks may be not critical meaningful. (2) FSL is the kind of learning task with limited data-scale. For a deeper network, it always has large number of parameters needed to be updated. In the data-limitation condition, too deep network could meet insufficient updating of parameters in backpropagation due to the too long backpropagation path. In parameter updating, shallower networks are more flexible, while the deeper networks look bulky. In short, it does not mean that deeper networks always outperform shallower networks. The size of network should match the specific task and data resources.

### 3.9. Compare with related works

In order to show the superiority of our method, we conducted several experiments to compare with some recent related researches. Argüeso et al. (2020) used Siamese Network, Triplet Network, and PV as their experimental material. They set a different data splitting: 32 classes are used for training and the rest six classes (apple four classes, blueberry healthy, cherry healthy) for testing. They listed results of three methods: transfer learning, Siamese Network, and Triplet Network. Their backbone network is Inception-V3. In order to be comparable, we executed the experiments with the same data setting as their work. Mini-ImageNet is used in base-training, 32 classes of PV are used in meta-learning, and the rest 6 classes are used in test. The results of e32–e34 are shown in Table 9.

**Table 9:** The results compared with related works.

**ID**	**Method**	**1-shot**	**5-shot**	**10-shot**	**20-shot**
		**Data setting in Argüeso et al. (** **2020** **)**			
	Finetuning (Argüeso et al., 2020)	18.2	25.4	30.3	41.1
	Siamese contrastive (Argüeso et al., 2020)	50.2	64.2	70.2	74.1
	Siamese triplet (Argüeso et al., 2020)	65.2	72.3	76.8	81.8
	Single SS (Li and Chao, 2021b)	74.5	89.7	92.6	93.9
	Iterative SS (Li and Chao, 2021b)	75.1	90.0	92.7	93.9
e32	**Ours MB**	76.4	91.0	93.2	94.2
e33	**Ours MB+CMSFF**	80.0	91.9	93.7	**94.3**
e34	**Ours MB+CMSFF+CA**	**80.4**	**92.8**	**94.1**	**94.3**
		**Data Split-1 of Li and Chao (** **2021b** **)**			
	Baseline (Li and Chao, 2021b)	32.8	46.7	64	73.2
	Single SS (Li and Chao, 2021b)	33.7	50.9	66.7	74.7
	Iterative SS (Li and Chao, 2021b)	34	53.1	68.8	75.6
e35	**Ours MB**	55.7	72.8	76.7	79.5
e36	**Ours MB+CMSFF**	60.6	**78.4**	**82.4**	84.3
e37	**Ours MB+CMSFF+CA**	**60.7**	78.1	82.2	**84.5**
		**Data Split-2 of Li and Chao (** **2021b** **)**			
	Baseline (Li and Chao, 2021b)	43.9	68.5	78.7	89.1
	Single SS (Li and Chao, 2021b)	44.7	74.7	85.7	89.7
	Iterative SS (Li and Chao, 2021b)	46.4	76.9	89.2	91.9
e38	**Ours MB**	77.1	91.1	92.9	93.8
e39	**Ours MB+CMSFF**	78.8	91.6	93.5	94.6
e40	**Ours MB+CMSFF+CA**	**79.1**	**92.2**	**94.0**	**95.1**
		**Data Split-3 of Li and Chao (** **2021b** **)**			
	Baseline (Li and Chao, 2021b)	50.7	63.1	77.2	89.3
	Single SS (Li and Chao, 2021b)	52.3	67.6	79.9	90.1
	Iterative SS (Li and Chao, 2021b)	55.2	69.3	80.8	91.5
e41	**Ours MB**	78.1	89.4	91.4	92.6
e42	**Ours MB+CMSFF**	80.6	90.8	92.4	93.3
e43	**Ours MB+CMSFF+CA**	**81.5**	**91.1**	**92.8**	**93.4**

(Ours: backbone network: Resnet12; distance metric: cosine similarity; base-training: Mini-ImageNet).

**Figure 7:**
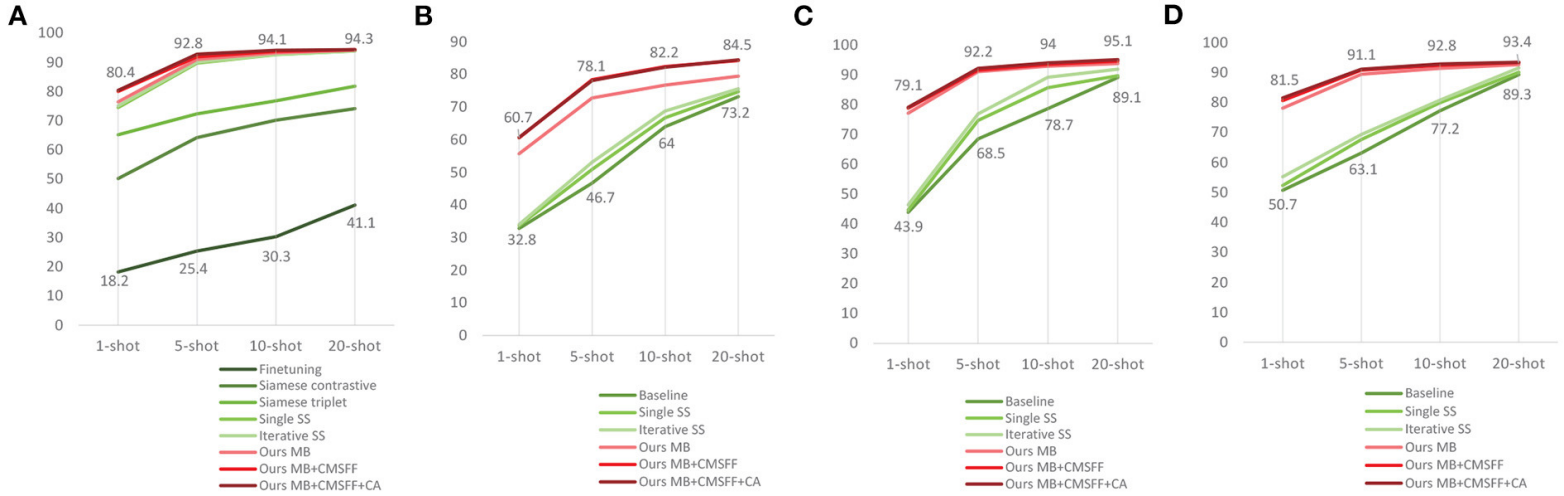
The results compared with related works. **(A)** Our work compares with (Argüeso et al., 2020) and (Li and Chao, 2021b). **(B)** Our work compares with Li and Chao (2021b) using the data split-1. **(C)** Our work compares with Li and Chao (2021b) using the data split-1. **(D)** Our work compares with Li and Chao (2021b) using the data split-1.

We also compared with Li and Chao (2021b). They proposed a Semi-supervised (SS) FSL approach. The baseline is a typical fine-tuning model. The Single SS adds Semi-supervised step on the top of baseline. The Iterative SS adds one more Semi-supervised step on the top of Single SS. PV was also used as their experimental material and set to three splits. Each split has 28 classes for training and the rest 10 classes for testing. They compared with Argüeso et al. (2020) too. We also conducted experiments by our methods with the same data settings as Li and Chao (2021b). The results of e35–e43 are shown in Table 9. All the comparison results are shown in Figure 7.

The data settings of the two references are different from our data settings. The results indicate that our method outperforms the existing works with all data settings, which means that our method is superior and robust.

## 4. Discussion

### 4.1. Motivation and contribution

The method learning from few samples is very promising in plant disease recognition, which has wide range of potential application scenarios for its saving of cost on data. When expanding the range of application, a well-established model of FSL can easily generalize to novel species or diseases without retraining and providing large-scale training data. However, some existing limitations of the FSL itself and the specific applied areas are needed to be considered. Our main contributions in this work are two-folds: (1) we propose to merge the CMSFF in the backbone network to enhance the feature representation, and combine the CA to focus on the informative channels; (2) we propose a group of training strategies to match the different generalization scenarios.

### 4.2. Limitation and future work

The theoretical research of FSL is in the stage of rapid development at present. Although FSL is very suitable for plant disease recognition, the applications of smart agriculture have just begun (Yang et al., 2022). In this research direction, there are still huge potential space needed to explore. In here, we discuss the limitations of this work and some future works.

**1. Multi-disease**. The PV and AFD used in this work as target data which have a common characteristic that only single disease is included in per image. In fact, once a plant is infected by the first disease, it is easily infected by other diseases because the immune system is attacked and becomes weak (Barbedo, 2016). Multiple diseases occur in a plant is more common in the real field condition. But the combinations of different diseases are too many to collect sufficient samples for each category from classification perspective (e.g., three diseases of a species generate 7 categories). The current researches prefer to solve this problem by semantic segmentation. We do not cover this challenging problem due to limitations of data resources in this work.

**2. Formulation of meta-learning data**. The samples of PV were taken under controlled condition (lab-settings), which have a clean board as the unified background, the illumination is under controlled, only single leaf in per image, only single disease occurs in per leaf. The settings are simple and very different from the in-wild conditions. That is the reason many researches already achieved high accuracy by using deep learning CNNs on PV (Hasan et al., 2020). But the samples of AFD were taken under in-wild condition, which have complex surroundings. When testing with AFD, we use PV in meta-learning, mainly considering that both datasets are about plant diseases. Since we did not find any other appropriate dataset, the degree of similarity of the data used in training and test was not taken in account.

According to our hypothesis, the degree of similarity of data used in meta-learning and test is higher, the adapting is easier, and the result would be better. It is demonstrated that the selection of meta-learning data is critical in this pipeline. The data used in meta-learning stage should be determined by the target. When the application scenarios cannot be predicted, how to formulate an appropriate meta-learning dataset is worthy to study. Inspired by Nuthalapati and Tunga (2021) and Li and Yang (2021), the effectiveness of a mixed dataset for meta-learning will be considered.

**3. Sub-class classification**. For the application of plant disease recognition, it is more meaningful to distinguish the diseases belonging to the same species. What farmers need more than anything else is a diagnostic assistant that can identify similar diseases belonging to the same plant. Although sub-class classification is difficult (Liu and Wang, 2021), it is an inescapable work in plant disease recognition and the performance is needed to be improved urgently. Fine-grained features of the lesions being the distinguishable features to solve this issue. In this direction, lesion detection and segmentation, fine-grained visual classification are involved.

**4. The quality and quantity of training data**. Most of the current researches of FSL deal with the configuration of data used in test, but very little work has concerned the data used in training. The common sense is that deep learning networks rely on large-scale data. However, a new direction is discussing the quality and quantity of training data recently (Li and Chao, 2021a,c; Li et al., 2021; Li Y. et al., 2022). These works indicate that part of data can achieve at the same performance as full data. Date quality can be assessed, which can guide to establish a dataset with enough diversity data while without redundant samples. The networks of appropriate depth using good data can achieve optimal results in many traditional CNN classification tasks.

In this work, we use large-scale data in base-training and meta-learning. The quantity of data follows the conventional settings for comparison purposes. The data quality assessment work is not involved in this work. For the specific topic of plant disease, the data quality is very important. We know that at different stages of development of plants and diseases, the symptom appearances are very different. How to construct a comprehensive set without redundant data to represent a disease is a valuable work in the future (Barbedo, 2018).

**5. Cross-domain**. The significance of cross-domain has been introduced in prior sections. We emphasize cross-domain again because it is common when we cannot predict the species, surroundings, and photo conditions in test. In this work, we consider it from training strategies. There are many aspects to explore in future work, such as network architecture, feature distribution calibration etc.

## 5. Conclusion

In response to the two problems when using FSL for plant disease recognition, we propose a network based on the MB approach that merges CMSFF and CA to obtain a richer feature representation. From experiments, we found that the CMSFF is effective to obtain richer feature representation, especially under the few-shot condition. The CA is an important compensation to the CMSFF, which helps to focus on these meaningful channels. Our method outperforms the existing related works, which indicates that our method is highly robust. The CMSFF+CA is an appropriate combination that fits for any algorithm that needs enhance the feature representation. In addition, a group of training strategies is proposed to meet requirements of different generalization situations. Many factors are discussed in this work, such as backbone networks, distance metrics etc. The limitations of this work and some new related research directions are discussed.

## Data availability statement

The dataset Mini-Imagenet for this study can be found in Kaggle, https://www.kaggle.com/datasets/whitemoon/miniimagenet; The dataset PlantVillage for this study can be found in Kaggle, https://www.kaggle.com/datasets/abdallahalidev/plantvillage-dataset; The dataset Apple Foliar Diseases for this study can be found in Kaggle, https://www.kaggle.com/c/plant-pathology-2021-fgvc8/data.

## Author contributions

HL, GP, RT, and S-KT: conceptualization. HL: methodology, experiment, and writing—original draft and editing. GP, RT, and S-KT: writing—review. Z-pQ: experiment and writing—review. All authors contributed to the article and approved the submitted version.

## Funding

This research was funded by the projects of Natural Science Foundation of China (Grant No. 12163004), the Yunnan Fundamental Research Projects (Grant No. 202101BD070001-053), and the Fundamental Research Projects of Yunnan Provincial Department of Education (Grant No. 2022J0496). This work was also supported in part by the Macao Polytechnic University—Edge Sensing and Computing: Enabling Human-centric (Sustainable) Smart Cities (RP/ESCA-01/2020).

## Conflict of interest

The authors declare that the research was conducted in the absence of any commercial or financial relationships that could be construed as a potential conflict of interest.

## Publisher's note

All claims expressed in this article are solely those of the authors and do not necessarily represent those of their affiliated organizations, or those of the publisher, the editors and the reviewers. Any product that may be evaluated in this article, or claim that may be made by its manufacturer, is not guaranteed or endorsed by the publisher.
